# The motivated appeal to hypocrisy: the relation of motivational threats to message rejection

**DOI:** 10.3389/fpsyg.2023.1253132

**Published:** 2023-10-20

**Authors:** David R. Pillow, Willie J. Hale, Janelle Kohler, Stephanie Mills, Jasmine Soler

**Affiliations:** Department of Psychology, The University of Texas at San Antonio, San Antonio, TX, United States

**Keywords:** hypocrisy, motivational threats, belongingness, consistency, control, self-esteem, trust, message persuasion

## Abstract

Few studies have focused on the conditions in which individuals perceive hypocrisy in others. The current study introduces and tests the Motivated Appeal to Hypocrisy (MAtH) hypothesis. This hypothesis examines core social psychological motivational threats and asks (a) whether these are related to the accounts of individuals in charging others with hypocrisy, and (b) whether these perceptions of hypocrisy are associated with reductions in the persuasiveness of persons targeted as hypocrites. Study 1 ( N=201) was based on qualitative coding of stories and revealed, as expected, that violations of core social motives involving belongingness, understanding, control, self-enhancement, and trust are involved in participants’ stories of hypocrisy. Study 2 ( N=237) used a multilevel correlational approach and demonstrated that violations of core social motives significantly predict perceptions of hypocrisy and the rejection of a person’s message or advice. The relation between social motive violations and message rejection was mediated by perceptions of hypocrisy.

## 1. Introduction

The appeal to hypocrisy is well known as a logical fallacy designed to counter the argument of an opponent. It is a form of an *ad hominem* argument that is used to discredit an opponent’s argument by asserting the opponent’s failure to act in accordance with their conclusion (“*Tu quoque*,” van Eemeren and Houtlosser, 2003). Unfortunately, the appeal to hypocrisy has become a staple of American life, with persons from both political parties constantly asserting inconsistency in their rivals’ behaviors, values, and use of rules. Consider, for example, the case of Senator Larry Craig who was arrested for disorderly conduct in 2007 after trying to initiate sexual conduct with another man in an airport restroom. Given that Senator Craig had a history of supporting anti-gay legislation that was arguably intended to control the behavior of others or make them outcasts, those on the left were quick to note the hypocrisy of Craig’s behavior (e.g., Saletan, 2007). Emphasizing that hypocrisy is in the eye of the beholder, numerous conservatives counter-argued that the episode was not hypocritical. Specifically, some defined hypocrisy narrowly by claiming that a hypocrite is one who does not truly believe their own message; Craig, they argued, believed his message, but merely suffered a moment of weakness (Jacoby, 2007; The New Republic Staff, 2007). This argument fits with empirical accounts of lay persons who regard temporary lapses of will as not necessarily hypocritical (Alicke et al., 2012), but the fact that liberals perceived the event as quite hypocritical suggests that these perceptions reside in the eye of the beholder via mechanisms that extend beyond definitional considerations.

The above story is consistent with what we call the motivated appeal to hypocrisy. Specifically, those that felt rejected or violated by the legislation were motivated to highlight the hypocrisy of the lawmaker as a means of discrediting the messenger and rejecting the message. Interestingly, however, previous research on motives and perceptions of hypocrisy is scant. Instead, most of the research in this area has focused on the schemas, and related conditions, that drive these perceptions of hypocrisy in others. Barden et al. (2005; see also Barden et al., 2014), for example, manipulated the order of information received and found that hypocrisy is more likely to be perceived when we first hear the message of a target individual and then learn of the inconsistent behavior than if the presentation order is reversed. Laurent and Clark (2019) replicated these results, finding that 84% of their respondents describe hypocrisy as having a temporal order where the attitudes manifest first followed by inconsistent behaviors. Alicke et al. (2012) manipulated information in a series of vignettes involving possible perceptions of hypocrisy—finding, for instance, that intentionally withholding information about previous behavioral inconsistencies leads the perceiver to label the target as more hypocritical.

Moving away from definitional schemas to consider the perceiver’s psychological state, Valdesolo and Desteno (2007) identified outgroup status as an important predictor of perceptions of hypocrisy, finding that individuals vary in their ratings of fairness of procedures leading to adverse outcomes. This is consistent with Kurzban’s (2014) argument that people are differentially selective in how they attend to information and build stories about themselves versus others, ignoring or explaining away inconsistent information about the self. The premise of our study is that such selectivity in story building is often associated with having experienced motivational threats when constructing one’s perceptions. Thus, rather than focus solely on the characteristics of a target, our research examines the interplay between characteristics of the target and their recollections of motivational violations as experienced by the perceiver in naturally occurring social interactions. This research does not purport to identify true instances of hypocrisy in target individuals. Rather, the objective of this research is to establish that the real-life stories of individuals and perceptions of hypocrisy, accurate or not, often involve perceived violations of core social motivational threats (Fiske, 2004). This descriptive and correlational research is designed to lay the foundation for the hypothesis described below.

### 1.1. The motivated appeal to hypocrisy hypothesis

We argue that claiming hypocrisy in another individual is a form of derogation that, more often than not, follows from a motivational violation and functions to aid perceivers in rejecting the message of a target. We refer to this as the Motivated Appeal to Hypocrisy (MAtH) hypothesis and argue that individuals are most likely to highlight inconsistencies in others (aka, “targets”) when attempting to dismiss a threat to the self. These threats (e.g., rejection, attempts at control, social comparisons that lower self-esteem) are often either based on the behavior or messages of a target that has implications for the self. In the psychological literature, this hypothesis follows partly from theoretical models positing that humans engage in motivated information processing to defend and maintain a positive self-image (Kunda, 1990; Lodge and Taber, 2000). We conceptualize the threats broadly and, as described later, borrow from Fiske’s (2004) work that identifies five basic motives driving social behavior and perception.

The MAtH hypothesis can be broken down into three propositions. The first is largely definitional and obvious: appeals to hypocrisy are a form of derogation. Consistent with this argument, we note that, linguistically, it is more natural to speak of “*charging* a person with hypocrisy” than it is to speak of “*perceiving* a person as a hypocrite.” The pejorative connotations of being labeled a hypocrite are strong. Literary evidence of this goes back to *Dante’s Inferno* (Alighieri, 2001) and passages in the Bible, (New International Version, 1984). Dante placed hypocrites on the next to last lowest level of hell, along with sorcerers, thieves, blackmailers, and seducers. Matthew 23:28 suggests that hypocrisy is akin to a crime as it reads, “So you also outwardly appear righteous to others, but within you are full of hypocrisy and lawlessness.” Modern empirical evidence regarding the degree to which hypocrisy has negative connotations is scant, though the word hypocrite is associated with terms such as liar, deceitful, phony, and two-faced (see Barden et al., 2005, 2014). In addition, Hale and Pillow (2015) found that stories of hypocrisy in others typically involve accounts in which targets charged with hypocrisy blame or derogate others or are perceived as pretentious. Finally, Jordan et al. (2017) found empirical evidence that hypocrites are disliked even more than liars because they use their hypocrisy to mislead others regarding their moral behavior.

The second proposition of the MAtH hypothesis concerns the antecedents leading to the perception or claim of hypocrisy and is this: individuals are more likely to perceive and highlight hypocrisy in others when they experience a motivational threat. No previous research has been reported in the literature directly examining this proposition; however, numerous studies have shown that violations of core social motives can lead to outgroup derogation (e.g., Florian and Mikulincer, 1998). Murrell and Jones (1993) found that when participants’ sense of control was manipulated, individuals were more likely to derogate others. There is also abundant evidence drawing from social identity theory (Tajfel and Turner, 1986) that individuals will derogate others when the motive to self-enhance is threatened.

The third proposition embedded in the MAtH hypothesis concerns the consequences of appeals to hypocrisy and is this: highlighting the hypocrisy of a target functions to reduce threats to the perceiver by enabling them to discount the values or message introduced by that target. Hypocrisy is a special case of derogation that occurs primarily when a target (who is the source of the message) is perceived as pushing a message and can also be identified as behaving in a manner inconsistent with said messages. This inconsistency allows the perceiver (aka, the message receiver) to attack the source for lacking credibility. Although we find no studies regarding the explicit role of *hypocrisy per se* in the persuasion process, there is a large literature on attitude change identifying likeability and credibility as central to persuasion (Chaiken, 1980; Petty et al., 1981; Benoit, 1987). In this literature, researchers typically manipulate the credibility of the source and treat persuasion as the dependent variable. The MAtH hypothesis is based on a similar logic but considers that the process can be reversed. It suggests that threats associated with an unwanted message will lead the receiver to search for and highlight evidence discrediting the source, thereby allowing the receiver to dismiss any threats associated with the message. Thus, this conceptualization treats credibility as a downstream, mediating variable that subsequently influences message rejection. Our research is designed to examine whether these propositions hold from a correlational perspective and thereby set the stage for future research.

### 1.2. Core motive threats and the perception of hypocrisy

Fiske (2004) proposed that our perceptions of, and interactions with, others involve largely five core social motives: belongingness, understanding, control, self-enhancement, and trust (BUCkET). Based on theory and previous pilot work, we hypothesize that evidence of threats involving this BUCkET of motives can be found in personal accounts of hypocrisy. Here, we briefly consider the role of each of these motives in accounts of hypocrisy from a theoretical perspective—starting with belongingness/rejection. Currently, there is no previous research supporting the specific proposition that the perception of being rejected increases claims of hypocrisy. However, Bourgeois and Leary (2001) reported that participants responded by derogating others when they experienced rejection and further found that derogating others helped participants maintain positive affective states. Similarly, rejection and ostracism have been linked to aggression in numerous studies (Williams, 2009; Rajchert and Winiewski, 2016). These studies make clear that threats to belonging lead to antagonistic responses—consistent with discrediting the messenger and messages involving rejection.

A second core motive is the need for understanding. Being able to understand ourselves and others serves both epistemic and pragmatic functions (Swann and Buhrmester, 2012) making one’s social world more meaningful and predictable. Consistency is a core feature of this form of understanding, and hypocrisy is, of course, often a claim of inconsistency. In short, acts of hypocrisy threaten our ability to make sense of a target’s behavior and can lead us to question the sincerity of their beliefs. Kreps et al. (2017) found that this is especially true when judging leaders who change their minds regarding a moral position. In fact, they found that unless the change of opinion was tied to a personally transformative experience, they were viewed more negatively and regarded as more hypocritical than someone who did not take a morals-based position. This indicates that people believe that moral stances should endure over time. Violating strong expectations of consistency may thus pose a threat that leads the perceiver to consider discrediting the message.

The third core motive noted by Fiske (2004) is the need for control. People work to maintain as many behavioral options as possible, desiring to feel that they control their outcomes. An abundant literature supports Brehm’s (1966) theory of psychological reactance in this regard—showing that individuals value a behavioral choice more after it is removed or restricted (Leotti et al., 2010) and that individuals will intensify efforts to regain control after a behavioral alternative is eliminated (Patall et al., 2008). We hypothesize that such efforts often include derogating the source of a message aimed at limiting a behavioral option by way of noting inconsistencies. Anecdotal examples of this phenomenon are not difficult to obtain. For instance, the teenager who is told by a parent not to text while driving argues back that dad is hypocritical because he talks on his cell phone while driving—noting that there is no difference. Additionally, Murrell and Jones (1993) reported that when participants’ sense of control was manipulated, they showed increased derogation of others (see also Laurent and Clark, 2019).

The fourth core motive concerns the need for enhancement of the self. Self-enhancement is largely synonymous with self-esteem, and a common tactic used in lab studies to threaten self-esteem involves providing participants with unfavorable social comparison information (Baumeister and Jones, 1978). As noted with respect to belongingness threats, Crocker et al. (1998) showed that individuals who experienced a self-enhancement threat exhibited higher ingroup favoritism and became prejudiced against outgroup members. Relatedly, threats to self-enhancement can occur based on how others who are close to them present themselves. As Jones and Pittman (1982) theorized, self-presentation tactics such as boasting, intimidation, and exemplification (i.e., attempting to appear as morally superior) all carry social comparison risks—with the latter of these specifically drawing possible charges of hypocrisy (Stone et al., 1997). Moreover, Alicke et al. (2012) found that the air of superiority evinced by hypocrites is an important factor in judgements of hypocritical behavior.

Finally, with respect to the motive of trust, Fiske (2004) notes that those who have been betrayed are hypersensitive to the negative behaviors of others. Betrayals of trust involve violations of expectations or social contracts. These expectations may be constructed either implicitly or explicitly, but either way, they typically involve behavioral standards that the perceiver expects the target to meet. For example, a person in a relationship may sometimes desire other sexual relations but avoids doing so because they trust their partner to hold to the same standard. When trust is broken, the individual is likely to question messages regarding such standards.

### 1.3. Current studies

The current studies were conducted to establish that the constructs specified in the MAtH hypothesis are correlated with one another. Specifically, we sought to determine (a) if stories of hypocrisy commonly involve motivational threats, (b) if motivational threats predict judgments of hypocrisy, and (c) whether judgments of hypocrisy predict a reduction in the persuasiveness of messages that are implicitly or explicitly communicated by the target. Study 1 explored the first objective; Study 2 was conducted subsequently to examine the second two propositions and is described later. Participants in Study 1 were asked to simply provide stories of hypocrisy from another person. We subsequently asked raters to read those stories and to code them with respect to themes of threats to belongingness, understanding (consistency), control, self-enhancement, and trust. We hypothesized that stories highlighting hypocrisy in others would contain elements of motivational threats more often than not (i.e., operationalized as greater than 50% of the time). We had no strong theoretical foundation for predicting which specific threats would be most frequent. Hence, this was left formally as an exploratory question. We did, however, anticipate that consistency would emerge as a frequent theme as it is foundational to some definitions of hypocrisy (though not all, see Hale and Pillow, 2015). We also expected self-enhancement threats to emerge frequently as (a) this threat arguably emanates from others behaving in a pretensive manner—where pretense is another common version of hypocrisy (Hale and Pillow, 2015), (b) hypocrisy was predicted as a specific self-presentational risk by Jones and Pittman (1982) to those behaving as morally superior to others, and (c) given prior evidence of a link between superiority and hypocrisy in vignette studies (Alicke et al., 2012).

## 2. Study 1

### 2.1. Methods

#### 2.1.1. Participants

Participants were students at a university in the southwestern United States who completed the study in exchange for partial completion of a course requirement. There were 279 (120 males, 136 females, 23 unidentified) initial participants. The average age of the participants was 19.29 years, *SD* = 2.82 years. Of those who elected to provide ethnicity data (257 of 279), participants were 44.76% Hispanic, 33.9% Caucasian, 8.2% African American, 8.6% Asian/Pacific Islander, and 4.7% mixed race/other. Fifteen participants were dropped because they did not respond to questions, and an additional 54 were excluded because their stories did not include personal stories of hypocrisy per instructions or were purely definitional (e.g., “Someone says one thing and does another”). Two stories were deleted as they were duplicate entries by participants leaving 210 stories for coding. Finally, 9 of the personal stories that were coded were not judged by both raters to have adequate evidence of hypocrisy (instead of settling ties, both raters had to agree), thus leaving 201 stories that form the basis for the analyses presented here. Participants in both Studies 1 and 2 were treated in accordance with APA ethical standards, provided informed consent, and all procedures were approved by the institutional review board.

#### 2.1.2. Procedure

Participants sat in a small room with six computer workstations. The first task associated with this study required participants to provide a typewritten example in which they recounted an episodic account of another person’s hypocritical behavior. Specifically, participants were instructed to do the following:

Please write an example of an instance in which you perceived that someone was a hypocrite or was behaving in a hypocritical manner. Please describe the incident in as much detail as possible, making sure to include your relation to the person, how the incident affected your perception of the person, and how the incident affected you.

After spending up to ten minutes composing their story, participants answered several questions assessing their reasons for selecting the incident they chose, as well as their impressions about the person in their story. Subsequently, additional questionnaires beyond the scope of the present study were collected.

#### 2.1.3. Coding

A coding scheme was developed, consistent with the major purposes of this study. Four coders were used, where 2 rated whether there was hypocrisy and the extent thereof, and 2 different coders coded whether or not there was evidence of specific psychological threats evinced in the story and the extent thereof. Importantly, the coders completed their tasks independently of one another, and in separate semesters. This was done to reduce experimenter biases that might artificially increase associations between judgments of threat and judgments of hypocrisy.

Two coders rated whether or not the story written by the participants were hypocritical, and if so, rated the extent of hypocrisy. Ratings were made using a 6-point scale ranging from 0 (not hypocritical) to 5 (extremely hypocritical), where 0 was treated as not including evidence of hypocrisy and scores of 1 to 5 were treated as including evidence of hypocrisy with scores indicating the extent to which the participant perceived the target as slightly hypocritical to very hypocritical. Given that the participants were instructed to provide a story that included hypocrisy, those stories rated by either coder with a 0 were considered unresponsive and were deleted—leaving the remaining ratings ranging from 1 to 5 when averaged across raters. This coding rule led to the removal of 9 stories from analysis as noted above. This limits the range of the data with respect to hypocrisy, but this issue is addressed in Study 2. The primary question of interest in Study 1 was primarily whether or not raters would find evidence of psychological motive threats in the story.

A separate set of coders determined, based on contextual information from the participants’ stories, whether and how many of the participants’ (or someone else’s) five core social motives were violated by the hypocrite. Coders again used a 6-point scale ranging from 0 to 5 to record their judgments with a 0 indicating that there was no evidence of the specific motive violation in question. In order to describe the frequency of motive violations, these motive violations were reduced to dichotomous variables where those scored 0 by both raters were judged to be not present (coded 0) and those judged to be present via the 1 to 5 scale were coded as present (1). There was also a catchall category so that coders could capture whether a particular story was merely definitional and/or had no evidence of violation of any of the core social motives. Coders were asked to consider various questions related to each core social motive when making their ratings. The full coding instructions are included in Supplementary Appendix A. By way of example, a sampling of these questions are as follows:

Belongingness violation: *Did the hypocrite’s actions weaken relationships with others in the story? Does the participant feel that the hypocrite caused others to feel rejected or excluded?*
Understanding violation: *Does the participant report shock or confusion over the actions of the hypocrite? Did the actions of the hypocrite make him or her less predictable to others?*
Control violation: *Was the hypocrite using or manipulating someone in the story? Did the hypocrite’s actions limit the autonomy of another person?*
Self-enhancement violation: *Did the hypocrite’s behavior undermine someone else’s self-esteem? Is the hypocrite portraying himself as superior (morally or otherwise) to others?*
Trust Violation: *Does the participant feel that the hypocrite cannot be relied upon? Does the participant indicate that the hypocrite betrayed someone else in the story?*

Two coders practiced rating subsets of 10 to 12 examples to establish inter-rater reliability (IRR). A total of 58 of the participants’ examples were used for practice. The coders were aware of the major goal of identifying violations of core social motives, but both were blind to the project’s specific hypotheses. High IRR was established among the two coders in training, obtaining an average Cohen’s κ of 0.80 across all of the categories and no less than 0.74 for any particular category.

Once high IRR was established using practice examples drawn from the total pool provided by participants, each of the two coders rated the remaining stories. After removing stories provided by participants that did not reflect episodic accounts of hypocrisy, both coders independently rated the same 201 stories. There was substantial agreement between the 2 raters across all threat categories and between the other 2 raters for hypocrisy ratings, and with respect to use of the rating scale, alphas were satisfactory. Percent agreements between the two raters for perceiving the presence of threats to belongingness, understanding, control, enhancement, and trust were 89, 80, 92, 81, and 90%, respectively. Using the full range of the ratings from 0 to 5, Cronbach’s alphas for the same respective threats were 0.86, 0.71, 0.88, 0.82, and 0.86. Also, an independent auditor, who is an author on this manuscript, reviewed all ratings and verified that the coders’ ratings were consistent with the coding scheme. When making judgments regarding the presence of hypocrisy for all 210 cases, 2 independent coders making these judgments agreed 96% of the time that hypocrisy was present (201 of 210 cases), and in judging the extent of hypocrisy present using the full 0 to 5 scale, a Cronbach’s alpha of 0.81 was obtained. Again, it is important to note that all participants were instructed to report examples of hypocrisy—thus restricting the range of possible responses on this variable. When scoring whether or not a threat or hypocrisy was present, both coders were required to agree.

#### 2.1.4. Data access

The data for this study, Study 1, and the data for Study 2 are available on the Open Science Framework at https://osf.io/u3mtw/?view_only=e01892e6ebca4cbfb0ce28b381b65a5d. The narratives provided for Study 1 have been edited to remove any names, specific locations, and dates mentioned so as to ensure that the stories are not identifiable except perhaps to those who wrote them. In addition, demographics have been stripped from the narrative data in order to ensure confidentiality.

### 2.2. Results

*Is there evidence of violations of the five core social motives present in participants’ stories of hypocrisy?* Supporting our primary hypothesis for Study 1, we found that participants provided evidence of violations of each of the five core social motives in their episodic accounts of another person’s hypocritical behavior. Specifically, coders agreed on the presence of at least one specific type of threat in all but 24 of the 203 stories given by participants (i.e., 179 participants or 88.2%). A *Z*-test for proportions confirmed that, as hypothesized, over 50% of the participants described motivational threats in their stories of hypocrisy, Z=10.22,p<0.001 . For the 179 stories judged to contain a threat, 32.3% included 1 threat, 28.9% included 2, 19.9% included 3, 6.0% included 4, and 1.0% included evidence of all 5 threats. One might question whether removing the threat to understanding would substantially change these results as it involves inconsistency—a threat that might be argued to be redundant with judgments of hypocrisy. This, however, was not the case. Removing the threat of understanding resulted in only 36 cases (17.9%) left being judged as lacking any motivational threat, where conversely, 82.1% of the cases were still judged as displaying evidence of threats to belongingness, control, self-enhancement, and trust.

Table 1 shows the percentages of violations of each core social motive in the participants’ stories. As can be seen, violations of the various motives were quite prevalent in participants’ stories, with each specific motive showing up in 24.9 to 46.3% of the participant’s stories, depending on the motive. Having already established at the person-level that most individuals recorded one of the five forms of threat in their stories, a chi-square goodness of fit analysis was conducted at the threat-level (where participant stories can contain multiple threats) to determine if some threats were present more or less than other threats (see Table 1). This analysis was based on judgments regarding the presence of a specific threat in each story, treating the judged absence of any motivational threat as a comparison. This resulted in 359 determinations of a threat taken from the stories of 201 participants (i.e., the average number of judged threats per person was 1.79), and we added to this number 24 cases with no threat present. As expected, these proportions were not equal, $\chi^{2}\left(5\right)=45.54,\;\;p<0.001.$ To determine the location of differences on a *post hoc* basis, pairwise comparison binomial tests were conducted using a Bonferroni correction where, $0.05/15_{pc}=0.003.$ Consistent with our predictions and the overall *Z*-test for proportions reported earlier, these tests revealed that all five threats were more prevalent than no reports of threat, all 5ps<0.003 . As for significant differences between types of threats, only one difference was obtained. Here, threats to understanding (24.3%) were significantly more prevalent than threats to control (13.1%), p<0.001 . Consistent with our expectations, threats to understanding occurred more frequently than other threats (24.3% of threats and occurring in 46.3% of cases)—contributing substantially to the chi-square lack of fit. Contrary to our expectations, threats involving self-enhancement were no more prevalent than other threats (18.3% vs. average of 18.88%). With respect to the relations between threats, coders saw violations of each of the motives as relatively independent or distinct, as evidenced by many small to moderate non-significant correlations amongst the motives ( $M_{r}=0.03$ ; see Table 1). Examples of violations of each of the core social motives in participants’ stories are as follows:

Belongingness violation: *“My best friend would always criticize me over the fact that I would hang out with my boyfriend too much and it seemed like I chose him over her. She would constantly hold some kind of grudge against me because of this. Her jealousy made it impossible for us to hang out with one another. However, once she got a boyfriend herself, she was nowhere to be found, constantly ditched her friends (including me), and would never return any of our phone calls unless her boyfriend was busy. I remember her always saying that me hanging out with my boyfriend so much was ruining our relationship, but here she was doing the same thing. Her hypocrisy has definitely made our relationship distant.”*
Understanding violation: *“There was an instance where a good friend of mine was talking to a group of mutual friends about this girl who was spreading rumors about her and talking trash behind her back, and how she (my friend) would never do that. Yet, my friend was doing the exact same thing she was accusing the other girl of doing. It made me feel confused because I really do not understand why someone, in this case my friend, would preach about someone stooping so low to talk trash and spread rumors then claim how much they do not like it, yet (in the same breath) do it themselves. How can they not see the irony in that? I dislike hypocrites very much.”*
Control violation: *“I often feel that my father is a hypocrite. He often says contradictory things when he is trying to control me and my siblings, and he treats my brothers much differently than he does my sisters. For example, he has told us many times not to say any curse words around him, when every other word that comes out of his mouth is inappropriate. He gets really mad at the girls if we curse, but hardly ever says anything to the boys. It makes me angry that he thinks he has the right to get mad at me when a bad word slips out of one of our mouths in front of him, yet he can openly curse in front of both of his parents. His behavior affects me because it makes me angry, and I believe that’s why I curse so much.”*
Self-enhancement violation: *“I had studied really hard with a friend for an exam hoping to receive a good grade, and I was able to come away with a grade that was acceptable to me. It was my best exam grade in that particular class and I was proud of myself, but my friend did not have the same view. She had received the same exact grade as me, and, not knowing what I had received on my test, texted me and told me what grade she received. I could tell that she was upset about the grade because she added the typical “frowning face” emoticon. I replied that she should not be upset because we both got the same grade, so we both should celebrate because we did well. To be nice, she congratulated me on my good grade, but went on to complain about her grade (which was the exact same grade as mine) and how it wasn’t a good enough grade and how she was expecting a MUCH better grade. This experience affected me negatively because she was one on hand telling me that my grade was good, but being a hypocrite by saying the same grade was bad for her. It made me feel inferior, as if she thought she was better or smarter than me. I did not really like that at all.”*
Trust Violation: *“My best friend wanted to know what was going between me and a certain guy I was interested in. She had her suspicions that he and I were romantically involved, but she was wrong. There wasn’t much to tell her and I told her so, but she just got offended and thought I was hiding stuff from her. She made a whole big deal about it. However, two weeks prior to this, she had a mini-relationship over the weekend where she met a guy, spent the weekend with him, and by Tuesday did not want to talk to him anymore…and I knew none of this at all. The only reason I found out was because I asked, so when she brought up me and this guy I was talking to I told her she was being a hypocrite for wanting to know all the details about my love life, yet failing to tell me much of anything about hers. I did not feel I could trust her anymore and I knew I would not be sharing any more important information with her. We are no longer friends.”*

**Table 1:** Percentages of motivational threats and inter-correlations.

Threat	Number of threats	Percent of cases	Belongingness violation	Understanding violation	Control violation	Self-enhancement violation
Belongingness	$71\;\;\left(18.5\%\right)$	35.3%				
Understanding	$93\;\;\left(24.3\%\right)$	46.3%	$\;0.17^{*}$			
Control	$50\;\;\left(13.1\%\right)$	24.9%	$-0.18^{**}$	−0.03		
Self-Enhancement	$70\;\;\left(18.3\%\right)$	34.8%	0.05	$\;0.14^{*}$	$-0.18^{*}$	
Trust	$75\;\;\left(19.6\%\right)$	37.3%	$\;0.23^{***}$	0.05	−0.06	0 .11
Zero	$24\;\;\left(6.3\%\right)$	11.9%				
Total	$383\;\;\left(100\%\right)$	-------				

N=201. Number of threats represents a count of whether or not each threat is judged to be present by both coders, with 358 present judgments made in 201 stories, with some stories containing more than one than one threat. The percent of cases is based on the number of threats divided by 201 and multiplied by 100. Phi-coefficients are displayed where each violation is treated as present (1) or not present (0), *p < 0.05, **p < 0.01, ***p < 0.001.

Supplemental analyses were conducted to determine if ratings of the 5 motivational threats predicted the *extent* of hypocrisy reported. Analyses were based on average severity ratings of the 2 raters who judged the extent of motivational threats and the average severity judgments of the 2 independent raters who judged the extent of hypocrisy. Overall, the motivational threats were found to account for significant variance in extent of hypocrisy with a multiple $R^{2}$ of 0.13, F(5,195)=5.98,p<0.001 . Extent of hypocrisy (using a 1 to 5-point scale) was predicted significantly by threats (using a 0 to 6-point scale) to belongingness (β=0.20,p=0.005) , control (β=0.25,p<0.001) , and self-enhancement (β=0.25,p<0.001) . Extent of hypocrisy was not significantly predicted by threats to understanding (β=0.05,p=0.50) or threats to trust (β=0.07,p=0.33) . The significance of these relations does not change at the level of zero-order correlations, and there were no signs of multicollinearity $\left(M_{VIF}=1.10\right).$ It is important to note, however, that the range of hypocrisy ratings was restricted in these data given all cases included stories where hypocrisy was judged to be present (consistent with participant instructions). Study 2 addresses these issues.

### 2.3. Discussion

Study 1 was designed primarily to determine if motivational threats could be detected within stories of hypocrisy. This was accomplished. As all stories were judged to involve hypocrisy, the association between the mere presence of a threat and the presence of hypocrisy (a constant) could not be estimated. This may explain why the extent of threats to understanding (or consistency) and trust were unrelated to judgments of the extent of hypocrisy, and why extent of threat perceptions only accounted for 13% of the variance in hypocrisy perceptions.

When providing stories of hypocrisy, participants were asked to discuss how the incident affected their perception of the hypocrite and how the incident affected them personally. These instructions likely influenced the type of person selected. In addition, the very specific instructions could have prompted participants to provide context (in the form of accounts of violated social motives) supportive of the accusation of hypocrisy, adding nuance to the episodic accounts that might not have been reported otherwise.

Another limitation is that our qualitative approach did not allow the participants themselves to indicate the degree to which they felt each need was violated and instead relied on third parties who lacked an affective connection to the emotionally charged content present in many of the stories. This could potentially result in underestimating the observed associations between motivational threats and perceptions of hypocrisy. On the other hand, the judgments of hypocrisy and their associations with threats could be an artifact of the expectation sets of the research assistants who conducted the coding—thereby resulting in the overestimation of observed effects. Study was designed to address these limitations.

## 3. Study 2

Several steps were taken to address the limitations present in Study 1 and to extend our arguments. First, we employed a multilevel, correlational study design where participants rated 6 individuals that they knew. The selection of these individuals was based on a series of prompts designed to yield a list of individuals that varied widely in their potential for both positive and negative evaluations. In contrast to Study 1, participants were not prompted to select target individuals based on their hypocrisy. Instead, the set of prompts included asking participants to think of persons who had violated specific core social motives (e.g., who had rejected them or violated their trust), as well as friends who, most likely, had not violated social motives. In this manner, Study 2 increased the range of responses for both the hypocrisy and motive violation variables. Secondly, Study 2 explicitly required participants themselves, rather than third party coders, to rate individuals on all variables of interest. Additionally, participants were not asked to select individuals who they perceived to be hypocrites; hence, subsequent ratings of hypocrisy could not be attributed to an activated schema regarding hypocrisy. Also, rather than using a single assessment of perceptions of hypocrisy, we used multiple items based on theoretically grounded definitions of hypocrisy drawn from the extant literature. Finally, we attempted to link perceptions of motive violations and hypocrisy to likelihood of rejection of someone’s messages, a form of derogation representing both an attitudinal and behavioral consequence theoretically related to our main study variables.

Using a multilevel regression design where the ratings of multiple targets were nested within participants, we hypothesized that (a) ratings of violations of core social motivational threats (i.e., belongingness, understanding, control, self-enhancement, and trust) would positively predict hypocrisy ratings, (b) ratings of hypocrisy would positively predict message rejection, and (c) violations of core motives would predict message rejection via indirect effects through hypocrisy. We did not make formal predictions regarding direct effects from motive violations to message rejection but allowed for them in specifying the model. Nor did we formulate strong hypotheses regarding the relative predictive strength of the motive violations, but we did revisit a corollary of the hypothesis specified in Study 1 regarding the frequency of self-enhancement threats. Specifically, we examined the possibility that self-enhancement threats would emerge as one of the stronger predictors of hypocrisy relative to other threats, consistent with Jones and Pittman (1982). In sum, where Study 1 demonstrated that motivational threats frequently appear in narrative stories of hypocrisy, Study 2 was designed to (a) examine the proposition that motivational threats predict judgments of hypocrisy, and (b) examine the proposition that these judgments of hypocrisy are associated with message rejection.

### 3.1. Method

#### 3.1.1. Participants

Participants were 237 students (85 males, 147 females, 5 unidentified) at a university in the southwestern United States who completed a questionnaire for partial completion of a course requirement. The average age of the participants was 19.19 years, *SD* = 2.93 years. With respect to ethnicity, participants were 45.3% Hispanic, 26.7% Caucasian, 11.6% African American, 15.2% Asian/Pacific Islander, and 0.9% other. Five persons skipped all demographic questions.

#### 3.1.2. Procedure

At the beginning of the study, participants were instructed to identify and name (using initials) 10 targets, five for each of the two groups of prompts. One set of prompts included target individuals who each explicitly violated one of five particular motives as follows: “a person who tries to control you;” “a person who has in some way rejected you or your values;” “a person who acts as if he/she is better than (or morally superior) to others;” “a person who you do not feel as though you can trust,” and “a person who is difficult to understand or predict.” The other set of prompts were written to generate five neutral (or motive irrelevant) targets and were selected to ensure variability with respect to ingroup/outgroup status and to generate at least some targets who were viewed positively. These prompts were as follows: “a friend of the same gender as you;” “a liberal;” “a conservative;” “a religious person,” and “an opposite sex friend.” The prompts were provided in alternating order (neutral then threat, and then repeating). After naming these targets, participants were instructed to rank the people in both groups (threat vs. neutral) separately with respect to how easy it was to think of the target with respect to their associated prompt. Following this, the experimenter selected 6 of the 10 target individuals so as to ensure that 6 distinct targets were selected with 3 generated from threat prompts and 3 from neutral, and, using a selection schedule, selected to provide approximately equal distributions of prompts across participants—though allowing participants to skip prompts for which they could not generate a target. Participants were then left in the computer room with their list of targets to complete all questionnaires. This procedure resulted in ratings of 717 targets generated with no explicit threats and 704 targets based on an explicit threat, where 94.4% of the participants generated and reported on 3 threat-prompted and 3 neutral targets. Only 1 participant responded to only 1 threat prompt, where the remaining 12 responded to at least 2. Participants completed a manipulation check item where they rated the person selected on the ease of generating a target for each prompt.

After the target individuals were identified, participants then answered items endorsing the degree to which the top three targets from each group violated their core social motives, the degree to which they viewed each target to be a hypocrite, and how likely they would be to accept advice or follow warnings from each target. Afterwards, they completed a series of measures assessing several dispositional and demographic characteristics. Reliability estimates for the scales reported below were obtained using multilevel analysis to partition within-person effects (assessing variability across targets) from between persons effects. Within-person reliability estimates were of major interest and comprise those reported.

#### 3.1.3. Measures

##### 3.1.3.1. Threats

Two items were written to assess each of the 5 threats, resulting in a total of 10 items. The number of items assessed per threat was kept low as participants provided these ratings for 6 target persons that they knew, thus providing a total of 60 ratings. Participants kept a sheet with them that listed Persons 1 through 6 and were asked to “please rate Person 1 (and later 2, 3…6) with respect to each of items below. Note that a blank has been left in each statement to refer to this person.” The extent to which these targets presented as threats was measured on 7-point Likert scales (1 = Strongly Disagree to 7 = Strongly Agree) and assessed threats to (a) belongingness (i.e., “I feel as though __ fully accepts me as I am;” “I often feel that __ rejects me or rejects values important to me.”), (b) understanding (i.e., “I feel as though I understand __ and can predict how he/she will be behave;” “I cannot really comprehend __‘s values or behaviors.”), (c) control (i.e., “I feel as though __ lets me live my own life the way I want to;” “I feel as though __tries to control the way I behave and wants to run my life”), (d) self-enhancement (i.e., “I feel as though __ is very humble and does nothing to put me down;” “__ tends to behave as though he/she is morally superior and/or better than me”), and (e) trust (i.e., “I trust __ has my best interests at heart;” “I cannot trust __ to treat me fairly”). Half of the items were written in the positive (aka, non-threatening) direction and were reversed coded. Reliabilities of these scales were 0.77, 0.60, 0.80, 0.64, and 0.80, respectively.

##### 3.1.3.2. Hypocrisy

Hypocrisy was assessed via 6 items. Participants rated their disagreement/agreement regarding descriptions of each target person as: a hypocrite; inconsistent; pretending to be something he/she is not; blaming (or criticizing) others when he/she does things just as bad or worse; choosing some values to uphold while being lazy about upholding other values; and failing to practice the very same thing he/she preaches. The first item employed the term “hypocrisy” itself, and the following items were generated based on the work of Hale and Pillow (2015) that explored 5 dimensions or types of hypocrisy. All items were assessed on 7-point Likert scales. The reliability for the scale was high (0.94).

##### 3.1.3.3. Message rejection

Four items were constructed to assess message rejection. For each target person, participants used to a 7-point Likert scale to report as follows: “I find that he/she is NOT persuasive;” “I find it easy to disregard his or her rules or values;” “I find it easy to dismiss his/her arguments,” and “I find that he/she inspires me to follow his/her ways.” The final item was reverse coded. Reliability of the items was found to be high (0.85).

### 3.2. Results

Analyses were conducted in two parts. The first set of analyses were conducted as a manipulation check to determine if the prompts were successful in producing variability as designed. The second set of analyses examined the patterns among the variables to test the specific hypotheses of the study.

#### 3.2.1. Manipulation checks

##### 3.2.1.1. Effectiveness of prompts

Tests were first conducted to determine whether the five prompts designed to prime memories of motivational threats had their intended effects. A random-intercepts, multilevel analysis with an unstructured covariance matrix was conducted with prompts nested within-persons, with analyses performed using mixed linear analyses in SPSS. *Prompts* served as the fixed factor which was specified as categorical with 10 levels (as there were 10 prompts). The analyses demonstrated that the prompts resulted in significant differences in the means, $F\left(9,\sim 1276\right)=113.50,\;\;60.38,\;\;114.26,\;\;1279.58,\;\;107.60,\;\;109.53,\;\;ps<0.001,$ for belongingness, understanding (consistency), control, self-enhancement, and trust, respectively.

**Figure 1:**
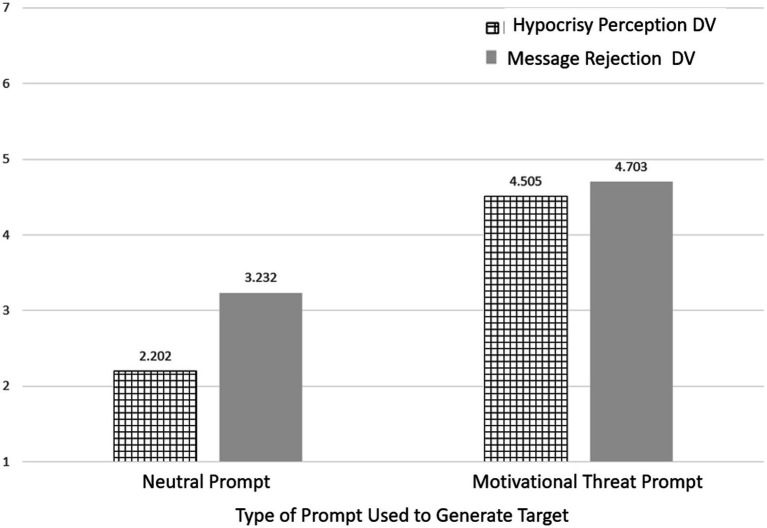
Mean differences on hypocrisy and message rejection ratings as a function of motivational threat prompts (*vs* neutral prompts).

Consistent with expectations, reports of specific motivational threats in targets were highest in response to those prompts with a similar threat. For example, the rejection (aka, belongingness) prompt resulted in the highest belongingness threat response compared to all other 9 prompts (though not always significantly higher). This pattern held for all 5 motivational threat prompts, thereby demonstrating that participants were attending to instructions and that the manipulation was effective (see Table 2 means that are bolded). Most importantly, the results support that construct validity was achieved as the questionnaire items designed to assess specific threat perceptions corresponded as expected to the prompts designed to induce those same threat perceptions. These patterns, examined using pairwise comparisons with Bonferroni corrections, are shown in Table 2. In addition, the mean differences in Table 2 show that the motivational threat prompts resulted in overall elevation of threat responses, hypocrisy ratings, and message rejection compared to the 5 non-threatening prompts. All of the means (i.e., perceptions of threat, hypocrisy, and message rejection) stemming from the BUCkET of *threat prompts* were consistently higher than the non-threatening prompts with the exception of inconsistency (aka, understanding)—where *ironically*, the mean differences were not consistently significant. To simplify key elements of this presentation, an overall multilevel comparison of means is shown in Figure 1—demonstrating that, on average, hypocrisy perceptions and message rejection were higher in response to motivational threat prompts compared to neutral prompts, F(1,1414)=649.82,p<0.001 , and F(1,1419)=300.05,p<0.001 , respectively.

**Table 2:** Means and standard deviations of motivational threats, hypocrisy ratings, and message rejection as a function of target person prompts.

Prompt	*N*	Reports of motivational threats					Person judgments	
		Belonging	Under-standing	Control	Self-enhancement	Trust	Hypocrisy	Message rejection
*A same sex friend*	226	$1.63_{a}$ (0.97)	$1.99_{a}$ (1.07)	$1.36_{a}\;\left(0.70\right)$	$1.75_{a}\;\left(1.04\right)$	$1.81_{a}\;\left(1.21\right)$	$2.01_{a}\;\left(1.32\right)$	$3.38_{a}\;\left(1.24\right)$
*A liberal*	94	$2.39_{bc}\;\left(1.52\right)$	$2.85_{b}\;\left(1.60\right)$	$1.91_{bc}\;\left(1.39\right)$	$2.74_{b}\;\left(1.80\right)$	$2.30_{a}\;\left(1.42\right)$	$2.82_{bc}\;\left(1.70\right)$	$3.45_{a}\;\left(1.59\right)$
*A conservative*	74	$2.36_{bc}\;\left(1.51\right)$	$2.60_{ab}\;\left(1.70\right)$	$1.97_{bc}\;\left(1.31\right)$	$2.76_{b}\;\left(1.78\right)$	$2.40_{ab}\;\left(1.71\right)$	$2.51_{ab}\;\left(1.76\right)$	$3.34_{a}\;\left(1.44\right)$
*A religious person*	143	$1.97_{ab}\;\left(1.40\right)$	$2.16_{a}\;\left(1.24\right)$	$2.00_{bc}\;\left(1.39\right)$	$2.05_{a}\;\left(1.49\right)$	$1.80_{a}\;\left(1.30\right)$	$1.99_{a}\;\left(1.43\right)$	$2.88_{a}\;\left(1.71\right)$
*An opposite Sex Friend*	180	$1.71_{a}\;\left(0.92\right)$	$2.51_{ab}\;\left(1.49\right)$	$1.54_{ab}\;\left(0.87\right)$	$1.83_{a}\;\left(1.21\right)$	$1.80_{a}\;\left(1.15\right)$	$2.17_{ab}\;\left(1.33\right)$	$3.17_{a}\;\left(1.49\right)$
*A person who has rejected you*	102	$4.92_{e}\;\left(1.57\right)$	$4.18_{cde}\;\left(1.78\right)$	$3.53_{d}\;\left(1.92\right)$	$4.73_{c}\;\left(1.96\right)$	$4.57_{d}\;\left(1.89\right)$	$4.49_{d}\;\left(1.43\right)$	$4.90_{c}\;\left(1.74\right)$
*A person who behaves inconsistently*	120	$2.80_{c}\;\left(1.52\right)$	$4.61_{e}\;\left(1.54\right)$	$2.32_{c}\;\left(1.42\right)$	$2.81_{b}\;\left(1.73\right)$	$2.94_{b}\;\left(1.84\right)$	$3.41_{c}\;\left(1.88\right)$	$4.12_{b}\;\left(1.60\right)$
*A person who tries to control you*	177	$4.15_{d}\;\left(1.75\right)$	$3.58_{c}\;\left(1.77\right)$	$4.87_{e}\;\left(1.56\right)$	$4.70_{c}\;\left(1.77\right)$	$3.88_{c}\;\left(2.09\right)$	$4.42_{d}\;\left(1.95\right)$	$4.24_{b}\;\left(1.80\right)$
*A person who acts as if better than others*	152	$4.21_{d}\;\left(1.68\right)$	$3.73_{cd}\;\left(1.72\right)$	$3.31_{d}\;\left(1.73\right)$	$5.72_{d}\;\left(1.33\right)$	$4.64_{d}\;\left(1.72\right)$	$4.90_{d}\;\left(1.79\right)$	$4.92_{c}\;\left(1.61\right)$
*A person who you do not trust*	153	$4.23_{d}\;\left(1.79\right)$	$4.19_{de}\;\left(1.77\right)$	$3.16_{d}\;\left(1.76\right)$	$4.73_{c}\;\left(1.76\right)$	$5.06_{d}\;\left(1.57\right)$	$5.08_{d}\;\left(1.63\right)$	$5.34_{c}\;\left(1.49\right)$

Standard deviations are displayed in parentheses below their respective means. All means are significantly different except those sharing a subscript, p<0.05 , with pairwise comparisons conducted using Bonferroni corrections. Means shown in boldface are those where threats prompted match reports of motivational threats. Higher scores indicate greater motivational threat or judgment.

##### 3.2.1.2. Easiness of recalling prompts

As noted earlier, participants answered a question regarding how it easy it was to generate a name (or recall a person) for each the 10 prompts. A within-subjects ANOVA was conducted to determine if participants differed in the ease of generating their list. Not surprisingly, significant differences were obtained with respect to ease of responding to the 10 prompts, F(9,2025)=69.91,p<0.001 . Briefly, participants found it easiest to think of a same gender friend, then an opposite sexed friend, and then a religious friend. Continuing from easiest to most difficult, they then identified individuals for four of the threats (control, self-enhancement, trust, and understanding), and then conservatives, liberals, and last, a person who had rejected them (see Supplementary Figure S1 for means).

#### 3.2.2. Hypothesis testing

**Figure 2:**
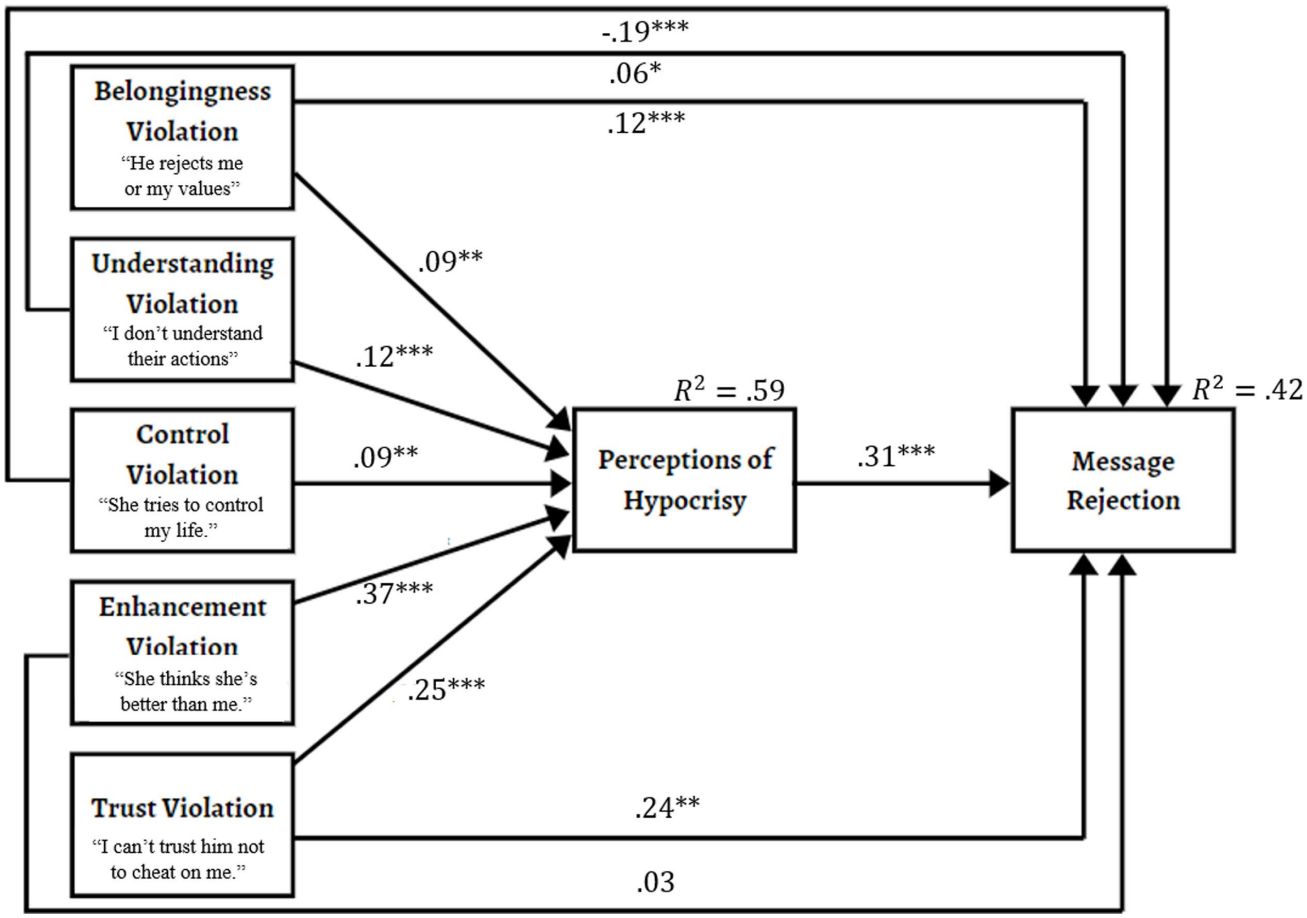
Predicting hypocrisy perceptions and message rejection as a function of motivational threats. Unstandardized, within-person path coefficients are displayed in the model—highlighting the partitioning of indirect effects through hypocrisy versus direct effects to message rejection. Correlations between predictors are not shown. Paths are estimated controlling for ease of target generation, and those effects are not shown. The paths from ease to hypocrisy and message rejection were 𝛾 = 0.05, *n.s.*, 𝛾 =−0.07, *p* < 0.05, respectively. * *p* < 0.05, ** *p* < 0.01, *** *p* < 0.001.

To examine the patterns of associations between variables, data were analyzed using MPLUS MSEM 1–1-1 multilevel mediation syntax adapted from Preacher et al. (2010). All hypotheses focused on within-person effects where individuals evaluated various target persons with respect to motive violations, hypocrisy, and message rejection. The fixed portion of the base regression model underlying this analysis can be written as, ${\hat{y}}_{ij}=\gamma_{00}+\gamma_{10}M_{1}+\ldots +\gamma_{50}M_{5}$ , where $M_{1}\;to\;M_{5}$ refer to the five motives, and ${\hat{y}}_{ij}$ refers to predicted values of perceived hypocrisy in multiple targets assessed across participants. The full model was estimated specifying two-level, random, MLR estimation, and included message rejection as a downstream outcome measure. Ease of generating each target evaluated was included as a covariate in the model predicting both hypocrisy and message rejection. The full model is shown in Figure 2.

Confirming predictions, violations of each of Fiske’s core social motives significantly and positively predicted hypocrisy in the within effects portion of the model, $R^{2}=.59,\;\;p<0.001$ (see Figure 2). Hypocrisy in turn predicted rejection of the target’s messages or advice, even as each motivational threat controlled for the remaining threats and ease, $R^{2}=.42,\;\;p<0.001$ . Target generation ease did not significantly predict hypocrisy, but was negatively related to message rejection, consistent with finding that friends were among the easiest to name as targets.

**Figure 3:**
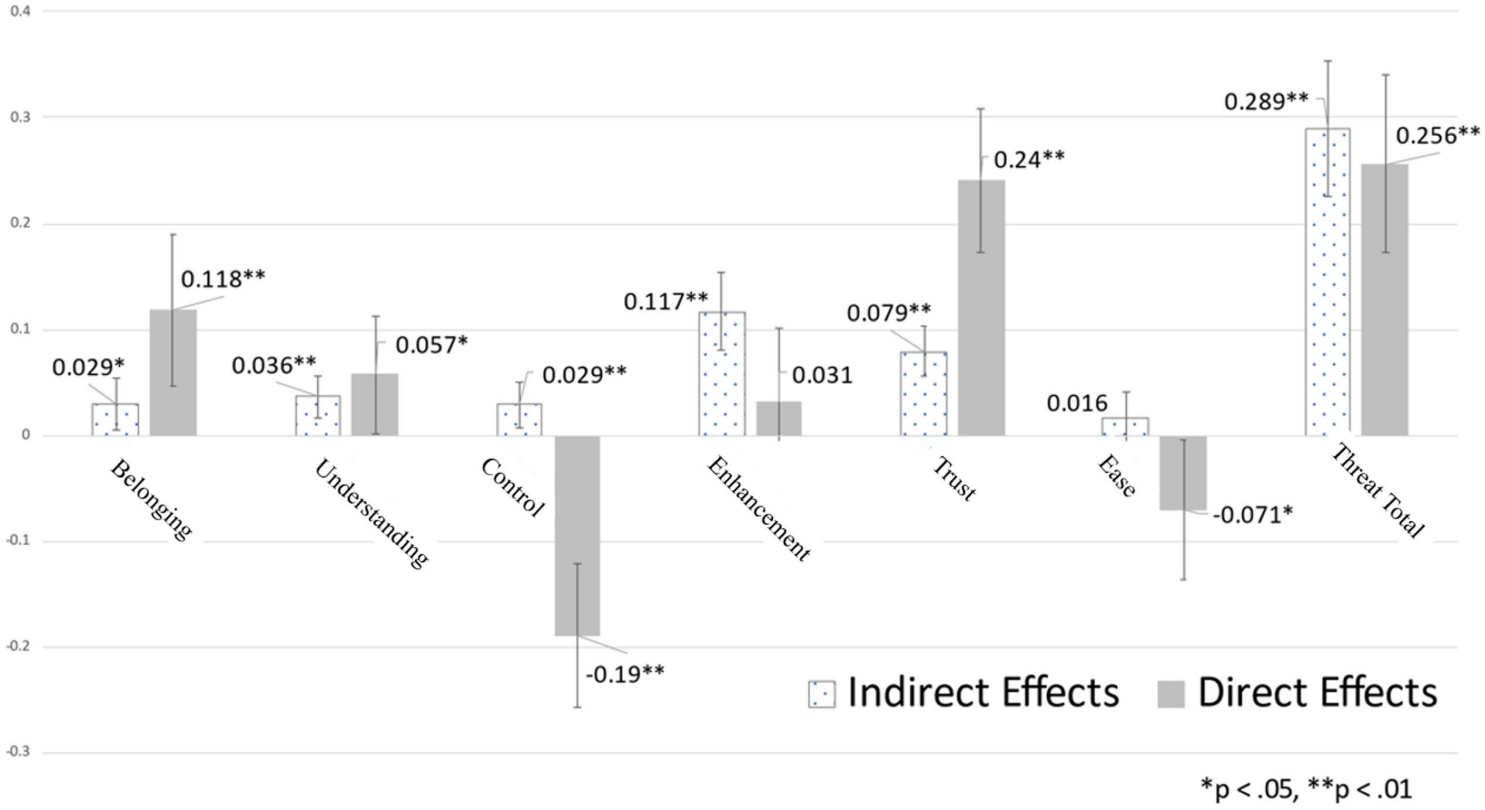
Indirect and direct effects of motivational threats and ease of target generation on message rejection. The indirect effects are from the listed variable to message rejection via *hypocrisy* ratings. The total threat effects are estimated controlling for ease of target generation, but only include the summative effects of the five threats from belongingness to trust.

Further analyses, displayed in Figure 3, partitioned effects into indirect effects and direct effects of threats predicting message rejection. Perceptions of hypocrisy significantly and positively mediated the relation between each of the five core social motives and the likelihood of message rejection. In addition, it was found that the combined indirect effects of threat on message rejection via hypocrisy was greater than the combined direct effects. As for direct effects, threats to belonging, understanding, and trust each significantly predicted greater message rejection. The indirect effect of self-enhancement threats (i.e., other’s portraying themselves as superior to the perceiver) to message rejection via hypocrisy was strong and resulted in no significant residual variance directly predicting message rejection. This strong relation is consistent with theoretical expectations. The only effect that was inconsistent with predictions concerns control violations. Though control positively predicted hypocrisy, its direct effect to message rejection was negative. As discussed later, this effect derives from a suppression effect that turns the bivariate relation between control violations and message rejection from a positive relation to a negative direct relation.

The model presented in Figure 2 specifies a particular direction of effects based partly on theory and partly on prompting individuals with respect to motivational threats. Specifically, our preferred model suggests that motivational threats lead to perceptions of hypocrisy, which then facilitates message rejection. That said, other directional effects are possible. We include in the Supplementary section a set of exploratory analyses (see Supplementary Figure S2) comparing alternative models, but full exploration of these models is beyond the scope of this paper, and we note that further work in this area is needed.

### 3.3. Discussion

Study 2 used a multilevel methodology where individuals evaluated multiple targets with respect to motivational threats, perceptions of hypocrisy, and degree to which the individuals would reject versus accept the messages of the targets evaluated. As detailed in the general discussion, the results support the proposition that motivational threats play an important role in perceptions of hypocrisy beyond the mere appearance of inconsistency. Moreover, the results are consistent with the proposition that perceptions of hypocrisy are positively associated with the likelihood of rejecting the message of a target.

## 4. General discussion

Our research demonstrates that lay stories of hypocrisy are strongly associated with perceptions of motivational threats. It further demonstrates a pattern of correlations that is consistent with the premise that thinking about motivational threats is associated with perceptions of hypocrisy, and, in turn, predicts rejecting messages. This pattern of associations is consistent with the Motivated Appeal to Hypocrisy (MAtH) hypothesis presented earlier, though alternative models needs to be ruled out in future research. The two studies provide insight into the specific nature of motivational threats associated with perceptions of hypocrisy. Study 1 identified specific threats using descriptive accounts provided by participants and rated by researchers with respect to Fiske’s BUCkET of motives; Study 2 used prompts generated by researchers to ensure variability among target persons with respect to threats to that same BUCkET of motives and was followed by the participants’ own evaluations of those target individuals. Using both of these methodologies, perceptions of hypocrisy were found to involve stories of motivational threats (Study 1) and to correlate with (Study 2) core motivational threats involving belongingness (i.e., the threat of rejection), understanding (i.e., the threat of not being able to comprehend the inconsistent behaviors of others), control (i.e., the threat of feeling coerced), self-enhancement (i.e., the threat of being directly or indirectly put down by others), and trust (i.e., the threat of violations to relationship agreements and expectations). Consistent with the MatH hypothesis, we obtained empirical evidence that individuals are likely to reject messages of those perceived to be hypocrites.

In many respects, the underpinnings and functions of the MatH hypothesis are perhaps obvious, but until now, have not been represented in the literature. Highlighting these effects allows researchers and theorists to appropriately construct a nomothetical net of perceived hypocrisy that includes motivational processes in addition to cognitive rules (Leotti et al., 2010) and intergroup conflicts (Beal et al., 2001; Barden et al., 2014). Our work builds on previous work that highlights that hypocrisy is a complex construct that is recognized in varying forms (Hale and Pillow, 2015)—for instance, it can involve seeing the irony that the blamer is also blameworthy and detecting pretentious self-aggrandizement in others. Consistent with Hale and Pillow (2015), this study found that most stories of hypocrisy included themes that extended beyond mere inconsistency.

Similarly, the present research demonstrates that the underpinnings of perceptions of hypocrisy take on forms easily missed by sampling from readily available anecdotes. For instance, it is natural to think of hypocrisy in politicians and preachers; however, our college student participants seldom mentioned such individuals and even found it difficult to think of people who are democrats or republicans. Instead, they focused on their interpersonal relationships. Here, trust emerged in our data as a potent breeding ground for MatH. Emerging adults spend much of their time and energies investing in these types of relationships (Lerner and Steinberg, 2009). These personal interactions involve negotiations regarding standards, implicit rules, and boundaries for relationship maintenance (Blumstein and Kollock, 1988). As these boundaries are often porous, it is easy for perceived offenses to occur—especially when generated as a function of the perceiver’s insecurities. When the aggrieved individual makes their implicit understanding of the rules explicit and notes a transgression, the accused individual is likely to question the rule and find fault and inconsistencies in the person expressing grievances.

Where relationship trust emerged as a strong predictor of hypocrisy perceptions, stories of perceived control were less prevalent than anticipated and demonstrated only small effects in predicting judgments of hypocrisy. This is surprising given that our predictions and assessment are rooted within the strong theoretical framework of psychological reactance (Brehm, 1966; Miron and Brehm, 2006). Examples of psychological reactance and hypocrisy are readily found in politics (e.g., when certain food items were eliminated from school lunches under Michelle Obama’s healthy eating plan, she was accused of being a hypocrite when pictured eating a burger), child relations with parents (e.g., parents are often accused of engaging in the same, or similar, risky behaviors that they instruct their children against), and at work (e.g., managers enjoy benefits not accessible to hourly employees). In general, individuals often construe rule makers who enjoy greater power as outgroup members and respond by dissembling and undercutting their power by noting their inconsistent use of standards (Lammers et al., 2010). Even though our participants were asked to identify persons who had attempted to control them, they were not explicitly asked to identify persons in power. Importantly, friends and lovers can attempt manipulations and control.

These findings deserve greater attention in future research, along with greater examination of the direct link between control violations and message rejection which, in our research, was negative. This is a curious finding and was not predicted. From a statistical perspective, the negative coefficient represents a clear suppression effect. In all possible models that we examined, control violations have a positive indirect relation via hypocrisy to message rejection. In addition, control violations, entered on its own as a predictor, relates positively to message rejection, but it has the weakest bivariate relation with message rejection of the threat variables explored here. Thus, the remaining residual direct effect turns negative when other variables are entered as predictors. Statistically, the direct effect also turns negative when controlling for other threat violations such as belongingness and self-enhancement threats. The substantive reason for this effect is unclear to us, but the explanation likely centers on the relatively lower zero-order correlation of control violations with message rejection. We speculate that this occurs because individuals may react more strongly to the idea of being controlled than to the message itself. For instance, individuals may respond negatively to feeling coerced by their employer to give donations while actually being supportive of donating to a cause (if done of their own free will). Of course, other explanations are possible and may be worth exploring in future research.

As stated in the introduction, Jones and Pittman (1982) specifically noted that exemplification (or, behaving as if morally superior) runs the risk of generating accusations of hypocrisy. As detailed below, our second study provides the first clear empirical evidence to support this long-held assertion—with the possible exception of work using scenarios involving preachers (e.g., Alicke et al., 2012). First, one of the two threat-to-enhancement questions explicitly went after exemplification by asking the participant to evaluate whether the target “tends to behave as though he/she is morally superior and/or better than me.” Second, the highest average perceived threat violation occurred with respect to the measure of self-enhancement in response to the self-enhancement threat prompt. Third, and most importantly, this threat measure was the strongest of the threats in predicting perceptions of hypocrisy.

Rejection has been linked to loss of belonging, meaning, control, and esteem (Williams, 2009). Moreover, Fiske (2004) argues that belongingness is a master motive. Consistent with our hypotheses, rejection was found to be a significant predictor of MAtH—serving as a mechanism to dismiss the relevance of the said rejection. This is consistent with Festinger’s (1954) work on social comparison processes which argued that we pay more attention to the opinions of others who are important to us. If the opinion has negative implications for the self, then denigrating the individual and reducing the importance of their opinion should reduce the threat. That said, threats to belongingness ranked relatively low in predictive power among the remaining threats. Again, we speculate that, as with perceived control, this may be because individuals are reporting on self-selected persons who are either relatively close to them—posing a small risk of rejection—or persons already considered outgroup members—and hence already considered irrelevant.

Finally, the inability to comprehend inconsistency was also found to predict hypocrisy. This should not be a surprise as the cognitive dissonance associated with hypocrisy is almost definitional. What is surprising is that the lack of understanding—assuming it is definitional—is not among the strongest predictors of hypocrisy. On the other hand, the work of Hale and Pillow (2015) provides potential insight here. Their qualitative work suggests that perceptions of hypocrisy in others most often goes beyond issues of apparent inconsistency to include narratives involving pretentiousness, bewilderment at how others assign blame in conflictual interactions, and other forms of indirect inconsistency (e.g., someone saying to not smoke cigarettes to others, yet chews tobacco). The quantitative results obtained here are, albeit indirectly, consistent with the findings of that previous work. Threats to self-enhancement stem from the pretentiousness of others, which here was found to have stronger effects than threats to understanding; threats involving trust stem from conflictual interactions where both sides have blame to spread and share. Thus, where inconsistency itself may provide the opportunity for others to see hypocrisy, our evidence indicates that motivational threats involving interactions with others have greater power in accounting for being perceived as a hypocrite.

### 4.1. Limitations and future directions

Although Study 2 was designed to produce variability in whether or not targets were perceived as hypocritical and included a broad range of possible targets, the participants’ responses focused more on personal relationships rather than prototypical accounts of hypocrisy often observed in the public (or political) realm. As mentioned earlier, this may be due to the sample age; but it may also result from the construction of the instructions and prompts as participants were instructed to write about how they were related to the target in their story and how their hypocrisy *personally* affected them. These instructions may have encouraged participants to write about hypocrisy within the context of personal relationships rather highlighting the public sphere. Future replications of this study with both older and more diverse populations as well as alterations in the instructions may be useful in exploring these possibilities. In addition, we caution the reader to note that this research focuses on self-reported data and examines only the perceptions of hypocrisy as experienced by the perceiver.

In addition, it is important to note that the predictive relations obtained here and pictured in Figure 1 are based on concurrent, correlational measures. Hence, causal linkages cannot be assumed. Future experimental research is needed in this area to determine whether threats to motivations precede perceptions of hypocrisy and message rejection or whether the motive to reject a message prompts the search for inconsistency in those promoting said messages. Multiple scenarios are possible. For instance, it is possible that seeing someone say one thing and then subsequently do another could result in bewilderment (or lack of understand) and lack of trust. Such a possibility suggests that the nature of the causal chain may depend on the nature of the specific threats. This remains an important direction for future research. In addition, future research should consider how motivational threats overlay with relevance as there is little reason to care that someone is hypocritical unless that person’s message is perceived to be self-relevant.

## 5. Conclusion

The current research highlights that motivational threats are associated with perceptions of hypocrisy. In doing so, it adds to a literature that has previously focused on cognitive factors and intergroup processes as explanatory factors. Moreover, the current research also serves to place these perceptions in the realm of the persuasion literature alongside issues of trust to more fully explain how individuals reject various types of messages and does so by drawing on intersections between psychology and philosophy with respect to understanding the use of heuristics in the process of human reasoning. Indeed, appeals to hypocrisy should not be considered valid forms of argumentation, but humans often respond to motivational threats in illogical ways. If we hope to remedy these errors in reasoning, it is important to understand the motivational factors associated with these perceptions.

## Data availability statement

The datasets presented in this study can be found in online repositories. The names of the repository/repositories and accession number(s) can be found at: https://osf.io/u3mtw/?view_only=e01892e6ebca4cbfb0ce28b381b65a5d.

## Ethics statement

The studies involving humans were approved by UTSA Human Subjects Institutional Review Board. The studies were conducted in accordance with the local legislation and institutional requirements. The participants provided their written informed consent to participate in this study.

## Author contributions

DP and WH led study conceptualization, measurement, data collection, analyses, and writing. DP serving as lead writer. JK and SM aided with analyses. SM aided with data coding and analyses for Study 1. JK aided with data analyses for Study 2. JK, SM, and JS aided with literature review, coding management, and editing. All authors contributed to the article and approved the submitted version.
