# Supplementary material for: The motivated appeal to hypocrisy: the relation of motivational threats to message rejection
Source: Front Psychol. 2023 Oct 20;14:1253132. doi: 10.3389/fpsyg.2023.1253132 (PMC10622961; doi:10.3389/fpsyg.2023.1253132)
Supplement: Supplementary file 1 [file Data_Sheet_1.PDF]

The Motivated Appeal to Hypocrisy:
The Relation of Motivational Threats to Message Rejection

SUPPLEMENTAL MATERIALS

David R. Pillow, Willie J. Hale, Janelle Kohler, Stephanie Mills & Jasmine Soler

The University of Texas at San Antonio

Supplemental Appendix A. Coding Methods, Materials, and Decisions

Coding Method

Narrative data are the definition of qualitative data (Syed & Nelson, 2015). However, as was the case in this study, it is often necessary to transform such qualitative data into quantitative data that can be worked with numerically. One team of people created the codebook needed for this transformation of data, while another team used the codebook to transform the data.

Coding Schema

Syed and Nelson (2015) postulate that deciding upon the unit of analysis is the first step on working with qualitative data. For this study, it was decided to rate each construct on a scale from zero to five; *not present* (0), *slightly present* (1), *somewhat present* (2), *present* (3), *definitely present* (4), *emphatically present* (5).

Saldana (2021) defines coding as a "heuristic, exploratory problem-solving technique without specific formulas or algorithms to follow." While coding may be heuristic, it is easier to begin with operational definitions of the constructs being examined. Operational definitions characterize codes in terms of observable properties (Chorney et al., 2015). In this case, clarifying questions were determined ahead of time based on the five core social motives (Fiske, 2014) and the five forms of hypocrisy (Crisp & Cowton, 1994; Hale & Pillow, 2015). To prepare for the creation of codebook created based on theory, coders read two articles, one on hypocrisy (Hale & Pillow, 2015) and one on the core social motives (Fiske, 2014).

These theories are the basis on which coding categories were developed and clarifying questions established. There was a category for each of the five core social motives; belonging, understanding, controlling, self-enhancing, and trusting (Fiske 2014). Five clarifying questions were developed for each of the core social motives. Coders referred to these questions during codebook creation. This allowed coders to reach consensus about the rating the responses should receive in terms of hypocrisy and the five core social motives when there were disagreements.

Coding Handbook Creation

Teams bring creativity to the coding process, but efforts must be taken to ensure coding results line up (Saldana, 2021). Because of this, a codebook is "the bedrock of a strong and successful coding system" and the next step in coding qualitative data (Syed & Nelson, 2015). The codebook) was developed using a theory-driven approach.

Four coders were assigned to develop a codebook and method for coding the data in a way that captured the level of hypocrisy or threat to core social motives. Coders consisted of three undergraduate students and one graduate student. Statements of hypocrisy collected from 165 psychology students attending a Southwestern university were used to train coders. These statements were only used for coding training, not the study results. Next, coders needed to become familiar with the data, in this case, the examples of hypocrisy given by participants (Syed & Nelson, 2015). During piloting, the coders found they agreed with Alicke et al. (2013) in that views of hypocrisy are dependent upon the attitudes of those doing the judging. Some participants gave examples coders did not consider hypocrisy. The following examples reflect participant statements of this type.

> *Example 1:* "The time I perceive that someone was being hypocritical to me was when my parents told me that Santa was not going to bring me presents this year because I was not being nice. However, I believe they were being hypocrite because Santa does not exist."

> *Example 2:* "My friend has stated time and time again that she hates cheese, nevertheless when it comes to cheesecake, she likes it."

> *Example 3:* "Parents usually tell you to not do something because it is wrong or bad yet they have done them themselves."

Some participants gave responses that were difficult for raters to come to a consensus on due to beliefs, experiences, and opinions influencing the level of hypocrisy or threat to core social motives seen. Coders worked together through discussing ratings for each response to discern their biases and learn to overcome them. These statements generally involved parenting, religion, and political issues. Some examples are given here:

> *Example 4:* "When my uncle told me that family is the most important thing and that I should take care of my parents in the future as well as I possibly could. He is an alcoholic and makes a lot of bad decisions. His family is suffering a lot because of his poor choices. I still love him because he is my uncle and I still take his advice because it is good advice regardless of who it came from. It doesn't affect me too much, but I am worried for his health."

Example 5: “I worked at a daycare in a Baptist church for 3 years I however am Catholic, to me it didn't matter because we all believe in the same God, we just worship in different ways. One of the older ladies that also worked in the daycare would often criticize my beliefs, even though we essentially believe in the same thing. She would only say things about my being Catholic to me but if someone else brought up the subject she would just act fine and happy with it as if she had no problem with it at all but then turn around and act completely different toward me.”

Example 6: “The "straight" women I know who are all about feminism and equality for women tend to treat me like shit because I'm not heterosexual, though I'm still a woman.”

Coders practiced coding data in small batches in a group at first, then individually, changing the codebook as necessary along the way. However, there comes a time when the coding manual must be considered finalized (Syed & Nelson, 2015). Once the coders achieved a high enough percentage of matching on the presence of each construct after use on a sample of practice statements the codebook was considered finalized.

Final Results Coding Training

Syed and Nelson claim there are three steps to training coders of qualitative data according to Syed & Nelson (2015). First, provide a copy of the coding manual to the coders. Discuss the manual and address any questions coders may have. For the second part of coding training a new group of three undergraduate Psychology students were recruited from a university lab and trained by one of the undergraduate students that helped develop the codebook, who was now a graduate student at the same university. The codebook was used to train the undergraduates and the previous set of practice statements were used to achieve a high enough match on if the construct was present or not.

Initially coders worked on small sets of questions together, coding out loud and discussing disagreements until consensus was reached. After a few rounds of group coding, coders assigned ratings to the practice statements of hypocrisy individually, still in smaller sections, to ensure consensus and acceptable interrater reliability. Once the coders agreed most of the time on the presence of either hypocrisy or the threat to core social motive, they were given the real data set to code. These ratings were used to calculate the final results.

Hypocrisy and Core Social Motives Coding Handbook For Coders

Because coding data is challenging and time consuming there are seven qualities a good coder should possess (Saldana, 2021). The first is organization. The second is perseverance. The third is ability to deal with ambiguity. Flexibility is the fourth trait of a good coder. The fifth is creativity. A sixth quality found in good coders is rigorous ethics. The final skill required to be a good coder is an extensive vocabulary.

You will be coding examples of hypocrisy given by study participants. Each statement will be coded for level of hypocrisy present in the eyes of the participant. Statements will also be coded for threats to each of the five core social motives. The following definitions and clarifying questions will assist in this task.

Definitions

For the purposes of this study, the following definitions will be used.

Hypocrisy- a feigning to be what one is not or to believe what one does not, behavior that contradicts what one claims to believe or feel. The false assumption of an appearance of virtue or religion. Sometimes the perceived hypocrisy originates in a presumption of superiority or personal values are judged to be hypocritical. Coding for hypocrisy requires you to step outside of your own personal values and beliefs to objectively consider whether the statement you are coding is an example of hypocrisy or not. Crisp and Cowton (1994) identified four forms of hypocrisy: pretense, blame, inconsistency, and complacency.

Core Social Motives-responsible for enhancing people's survival in social situations and offering a unifying framework for understanding the field of social psychology. (Note that students also read key passages from Fiske (2004) to better understand the threats and these readings were discussed in lab sessions.)

Belonging- the idea people need close social ties to other humans.

Understanding-conveys the importance of understanding one's environment to assist in predicting future happenings as well as making sense of what has passed. This understanding and ability to predict is a driving force behind attraction as well as prejudice against dissimilar others.

Control-allows people to feel effective in dealing with the world. It is a contingency between behavior and outcomes.

Self-enhancement-is preserving self-esteem or even more ideally, raising it.

Trusting-facilitates attachment and interdependence in close relationships.

Clarifying Questions

These questions are meant to help you discern the level of hypocrisy and threats to the core social motives in each given scenario. Every clarifying question does not need an affirmative answer, but some may be answered yes so emphatically that the score is dictated by the response to that individual question. This depends on the emotional content of the statement as well as the words used to describe the hypocrite in the scenario.

Hypocrisy Clarifying Questions

When coding for hypocrisy, code using the following scale: *not present* (0), *slightly present* (1), *somewhat present* (2), *present* (3), *definitely present* (4), *emphatically present* (5). Use the following questions to determine what value to assign each statement.

1. *The perceiver would describe the target as a hypocrite.*
2. *The perceiver would describe the target as inconsistent.*
3. *The perceiver would describe the target as pretending to be something that he or she is not.*
4. *The perceiver would describe the target as blaming or criticizing others when he or she does things that are just as bad or worse.*
5. *The perceiver would describe the target as choosing some values to uphold while being lazy about upholding other values.*
6. *The perceiver would describe the target as failing to practice the very same thing that he or she preaches.*

Belongingness Violation Clarifying Questions

When coding for belonginess violations, code using the following scale: *not present* (0), *slightly present* (1), *somewhat present* (2), *present* (3), *definitely present* (4), *emphatically present* (5). Use the following questions to determine what value to assign each statement.

1. *Did the target's actions weaken relationships with others in the story?*
2. *Does the participant feel that the target caused others to feel rejected or excluded?*
3. *Did the target's actions lead another to want to distance themselves from the perceiver or the group?*
4. *Did the target's actions threaten the stability of a close relationship?*
5. *Is the target using derisive, derogatory, or demeaning language about the perceiver or the perceiver's group?*

Understanding Violation Clarifying Questions

When coding for understanding violations, code using the following scale: *not present* (0), *slightly present* (1), *somewhat present* (2), *present* (3), *definitely present* (4), *emphatically present* (5). Use the following questions to determine what value to assign each statement.

1. *Does the participant report shock or confusion over the actions of the hypocrite?*
2. *Did the actions of the hypocrite make him or her less predictable to others?*

3. *Does the story highlight inconsistency that bothers the perceiver?*
4. *Does the perceiver seemingly describe disruptions to their world-view given the behavior of the target?*
5. *Does the perceiver talk about difficulty in understanding the target?*

Control Violation Clarifying Questions

When coding for control violations, code using the following scale: *not present* (0), *slightly present* (1), *somewhat present* (2), *present* (3), *definitely present* (4), *emphatically present* (5). Use the following questions to determine what value to assign each statement.

1. *Was the target using or manipulating someone in the story?*
2. *Did the target's actions limit the autonomy of another person?*
3. *Is the target (acting as an authority figure) limiting the options of the perceiver?*
4. *Does the target have unreasonable expectations of the perceiver?*
5. *Does the target make the perceiver feel trapped or helpless?*

Self-enhancement Violation Clarifying Questions

When coding for self-enhancement violations, code using the following scale: *not present* (0), *slightly present* (1), *somewhat present* (2), *present* (3), *definitely present* (4), *emphatically present* (5). Use the following questions to determine what value to assign each statement.

1. *Did the target's behavior undermine someone else's self-esteem?*
2. *Is the target portraying himself as superior (morally or otherwise) to others?*
3. *Is the person behaving in pretentious manner that annoys the perceiver?*
4. *Is the perceiver intimidated by the target?*
5. *Does the perceiver feel as though the target is putting them down in order to look better?*

Trust Violation Clarifying Questions

When coding for trust violations, code using the following scale: *not present* (0), *slightly present* (1), *somewhat present* (2), *present* (3), *definitely present* (4), *emphatically present* (5). Use the following questions to determine what value to assign each statement.

1. *Does the participant feel that the hypocrite cannot be relied upon?*
2. *Does the participant indicate that the hypocrite betrayed someone else in the story?*
3. *Does the perceiver feel that the target cannot be trusted?*
4. *Does the perceiver feel exploited by the target?*
5. *Does the target go back on their word or violate an informal contract?*

Data available

As referenced in the paper, two data sets are available. The first data set is for Study 1. It includes the redacted statements of the students who participated in the open-ended study providing stories of hypocrisy. It also includes the ratings of the 4 coders for each threat and for hypocrisy. Rather than settling ties, we took the conservative approach of only counting threats or hypocrisy when both raters agreed that it was present. This was done for any analyses that presents counts or percentages. For analyses that focus on extent of relations between variables, we took the average of the ratings.

The second data set is in long form and constitutes the data used for all multilevel analyses involving manipulation checks and that presented in Figure 1, estimation of the full model displayed as Figure 2, estimation of indirect and direct effects displayed in Figure 3, and estimation of model comparisons shown in the supplemental materials.

Additional materials can be obtained by contacting David Pillow at david.pillow@utsa.edu.

References

Alicke, M., Gordon, E., & Rose, D. (2013). Hypocrisy: What counts? *Philosophical Psychology*, *26*(5), 673–701. https://doi.org/10.1080/09515089.2012.677397

Chorney, J.M., McMurtry, C. M., Chambers, C. T., & Bakeman, R. (2015). Developing and modifying behavioral coding schemes in pediatric psychology: A practical guide. *Journal of Pediatric Psychology*, *40*(1), 154–164. https://doi.org/10.1093/jpepsy/jsu099

Crisp, R., & Cowton, C. (1994). Hypocrisy and moral seriousness. *American Philosophical Quarterly*, *31*(4), 343–349.

Fiske, Susan T. (2014). *Social beings: Core motives in social psychology* (3rd ed.). John Wiley & Sons.

Hale, W. J, Jr., & Pillow, D. R. (2015). Asymmetries in perceptions of self and others' hypocrisy: Rethinking the meaning and perception of the construct. *European Journal of Social Psychology*, *45*(1), 88–98. https://doi.org/10.1002/ejsp.2064

Saldana, J. (2021). *The coding manual for qualitative researchers* (4th ed.). SAGE Publishing.

Syed, M., & Nelson, S. C. (2015). Guidelines for establishing reliability when coding narrative data. *Emerging Adulthood*, *3*(6), 375–387. https://doi.org/10.1177/2167696815587648

Supplemental Figure 1

Ease of Target Generation, Listed by Target Prompt

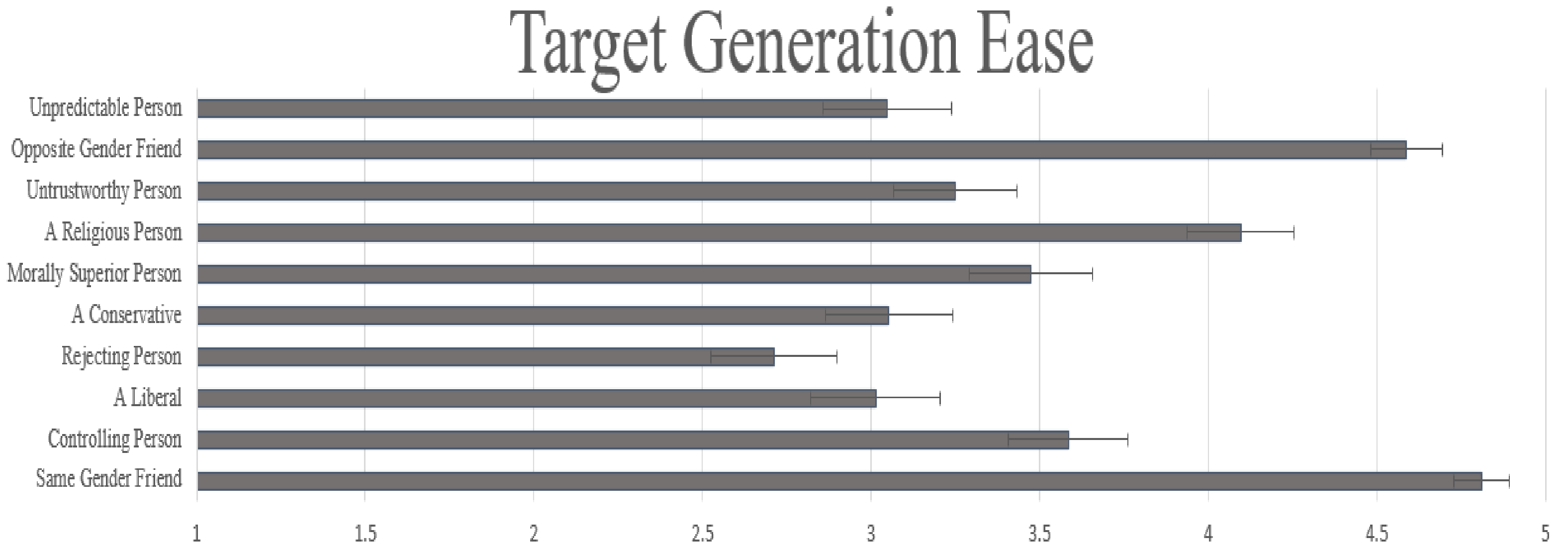

Note: Means and standard errors are displayed. The scale ranged from 1 (Extremely difficult/Impossible to think of someone) to 5 (Extremely Easy to think of someone).

Supplemental Figure 2

Directional Model Comparisons of Mediational Processes

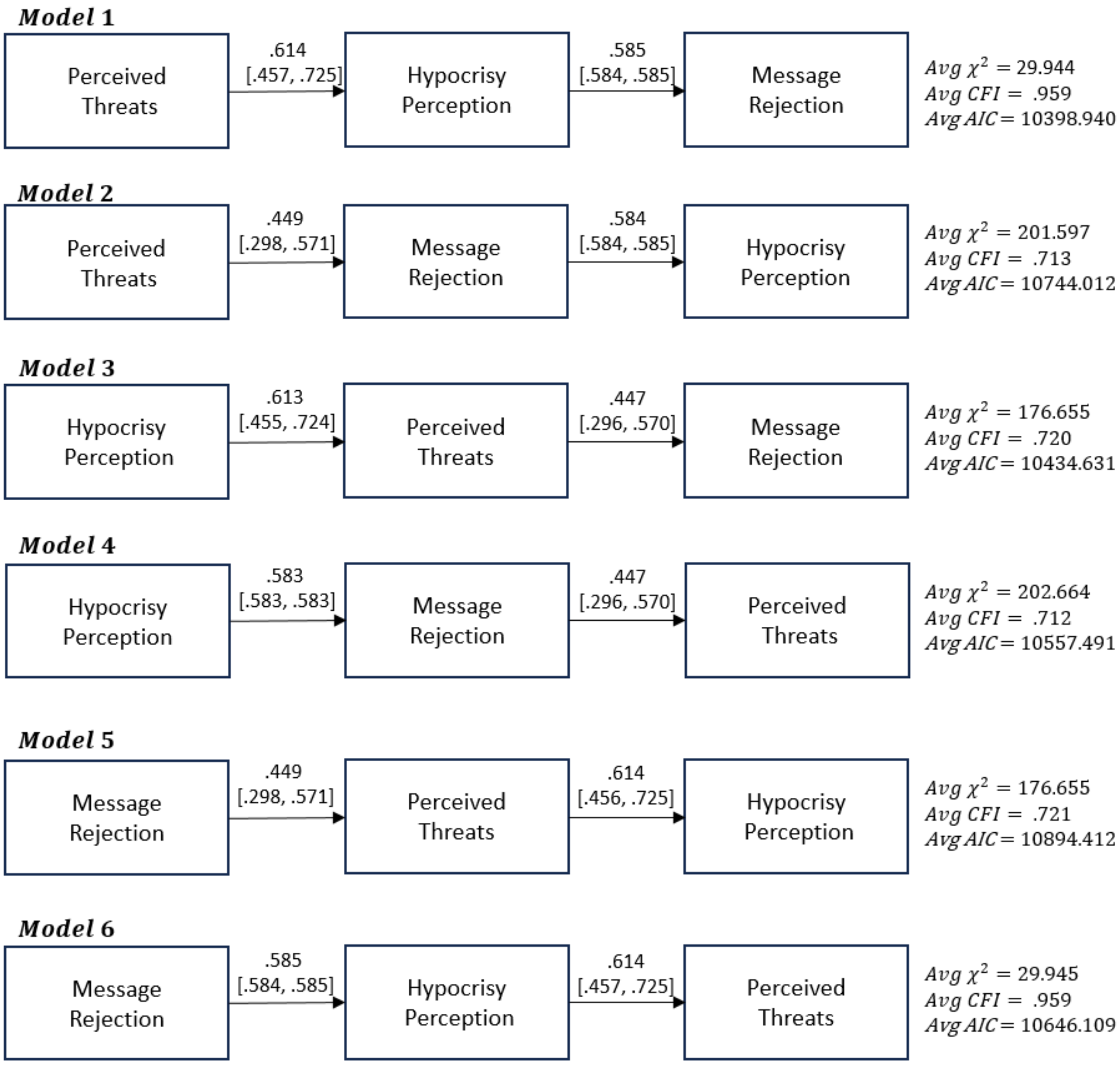

Note. Six models were compared following a X→M→Y pattern, with X referring to the exogenous predictor, M referring to the mediator, and Y referring to final outcome variable. Each model excludes the direct path from X to Y to estimate the extent of model misfit. The models as shown are averaged across the 5 threats. Thus, each model displayed was run 5 times, and then we averaged across parameter estimates and fit indices. The gammas (as this was multilevel) displayed are the averages and the values in parentheses represent the low vs high estimate. This range is always larger for paths involving perceived threats as that represents the key variable traded out on each run. The results show that Model 1 (our preferred theoretical framing) and Model 6 are indistinguishable, and fit the data better than models 2 through 5. This does not negate the possibility that other paradigms might show different patterns.

Supplemental Table 1

Model Fit Comparisons

Model Comparisons		3 Variable Models Using Specific Threats One at a Time				
		Belong	Understand	Control	Enhance	Trust
Model 1:	X^2	30.622	24.798	2.073	19.123	73.105
Threat→Hyp→MR	*CFI*	.959	.953	.998	.977	.909
	AIC	10353.711	10783.994	10575.811	10059.965	10221.221
Model 2:	X^2	216.395	83.953	223.202	265.312	219.123
Threat→MR→Hyp	*CFI*	.701	.837	.643	.663	.723
	AIC	10597.39	10890.399	10984.18	10701.135	10546.954
Model 3:	X^2	152.528	260.813	247.875	127.276	94.785
Hyp→Threat→MR	*CFI*	.791	.488	.602	.839	.881
	AIC	10280.366	10742.114	10636.262	10306.727	10207.686
Model 4:	X^2	221.733	83.953	223.201	265.312	219.123
Hyp→MR→Threat	*CFI*	.696	.836	.642	.662	.723
	AIC	10449.458	10481.284	10585.464	10770.624	10500.624
Model 5:	X^2	152.528	260.813	247.875	127.276	94.785
MR→Threat→Hyp	*CFI*	.792	.490	.604	.839	.881
	AIC	10741.192	11201.957	11096.599	10766.512	10665.802
Model 6:	X^2	30.622	24.798	2.073	19.123	73.107
MR→Hyp→Threat	*CFI*	.959	.953	.998	.977	.909
	AIC	10536.142	10834.722	10637.432	10589.239	10633.010

Note. Hyp = Hypocrisy, MR = Message Rejection. All X^2 *df* for the models using specific threats one at a time are 1.
